# Differences Between Girls and Boys in the Disclosure of Sexual Violence

**DOI:** 10.1177/08862605231221283

**Published:** 2024-01-22

**Authors:** Johanna Hietamäki, Marita Husso, Tiia Arponen, Hanna-Mari Lahtinen

**Affiliations:** 1 Finnish Institute for Health and Welfare, Helsinki, Finland; 2 Faculty of Social Sciences and Business Studies, University of Eastern Finland, Kuopio, Finland; 3 Faculty of Social Sciences, Tampere University, Finland; 4 City of Lappeenranta, Lappeenranta, Finland; 5 Faculty of Social Sciences, University of Helsinki, Finland; 6 School of Educational Sciences and Psychology, University of Eastern Finland, Joensuu, Finland

**Keywords:** child sexual violence, child sexual abuse, child victim survey, gendered violence, disclosure

## Abstract

This article addresses the differences between girls and boys in the disclosure of sexual violence. The dataset combines data from the Finnish Child Victim Survey (FCVS) of 2008 (*N* = 13,459) and 2013 (*N* = 11,364), focusing on victims of sexual violence, ages 11 to 17 years, from the perspectives of disclosure and gender. Frequency and percentage analysis, cross tabulation, and a Chi-square test were used in the analysis. In the FCVS for both years, around 85% of the victims were girls. In almost two-thirds of the cases, the offender was a relative, friend, or some other known person, while in more than one-third of the cases, the offender was unknown to the victim. The second most common case was that the victim knew the offender, who was not, however, a friend. Sexual violence was found to be, in many ways, gendered. Most of the victims were girls, and most of the offenders were men. There was also a gender difference in the disclosure of experiences. Twenty-one percent of the girls and 45% of the boys reported that they had not told anyone about their experiences. Irrespective of the type of offender, the victims most often (63%) told a peer about their experiences, while 23% told parents, and only 10% told authorities. Moreover, victims reported shame and fear, distrust toward adults, and disbelief that disclosure would be helpful as reasons for not disclosing their experiences. To address this problem, raising awareness of the phenomenon, promoting an atmosphere that supports disclosing experiences of sexual violence, and improving readiness to address them are required.

## Introduction

Not until the 1980s did sexual violence against children begin to be publicly addressed in Finland. The discussion originated with the identification of physical violence against children in health care (Laitinen, 2004, pp. 28−30). In recent years, the introduction of themes related to violence in the media and public discussions resulting from the MeToo movement have also contributed to rendering sexual violence against children visible and identifying it as a problem. The surfacing of sexual offenses against minors, particularly in 2018 and 2019, have highlighted such incidents as a social policy issue and a general topic of discussion, both in everyday life and in the media. For example, the harassment, grooming, and abuse of children in digital media have raised concerns (Save the Children Finland, 2021). Recently, several cases of violence reported by children have led to the detection of serial sexual offenders targeting children. In one case, a man had 134 victims. A parent found a conversation on her 9-year-old’s phone in which the child was talking to friends about the suspect (Mäentausta, 2021, Roiha & Kaski, 2022). Young people were overheard talking about another case at school, revealing 156 victims (Hankaniemi, 2022).

The number of cases reported to the police has increased six to eight times since 1980, reaching 2,120 in 2018 (Kaakinen et al., 2020). However, not all offenses come to the attention of the authorities. The majority of sexual violence remains known only to the victim and the perpetrator, which is why only a small proportion of sexual offenses appear in crime statistics (Bottoms et al., 2016; Lahtinen, 2022; Lahtinen et al., 2022; Priebe & Svedin, 2008). Due to limitations related to official statistics, self-report surveys are considered to be better measures of the prevalence of violence in general (Ellis et al., 2010).

Studies have shown that the consequences of sexual violence for children are diverse and wide-ranging. They are linked to various physical and psychological problems, such as chronic pain, inflammation, anxiety, depression, eating disorders, and post-traumatic stress (Amado et al., 2015; Broman-Fulks et al., 2007; Chen et al., 2010; Maniglio, 2009; Paras et al., 2009). Methods that consider the vulnerable situation of children in the investigation of child sexual violence (CSV) and assault are currently under development (e.g., the Barnahus project in Finland) (Laajasalo et al., 2022; 2018). However, current reports and guidelines do not particularly focus on how to promote the reporting of CSV to adults, nor do they provide information or discussion on the topic in schools (see the Lanzarote Convention). There is also no national 24/7 helpline aimed at victims of CSV or other forms of violence. Nevertheless, many phone and virtual services for children and adolescents have been established by NGOs in Finland.

To understand the phenomenon and to promote change in society, it is important that sexual violence is disclosed. If the experiences of CSV victims remain unreported to authorities, their scope will not be understood, and children damaged by the abuse and its repercussions will be deprived of assistance, support, or justice (Bottoms et al., 2016; Pipe et al., 2007). This will remain the case as long as the prevalence of CSV and its severity remain hidden as a social phenomenon and children’s experiences of CSV remain unrecognized.

## Sexual Violence Against Children

In public discussions, CSV has been identified as a social and child welfare problem and a crime that requires societal intervention guided by formal agreements and legislation. Nations such as Finland, which have ratified the UN Convention on the Rights of the Child, are committed to protecting children from all forms of sexual exploitation and violence and to taking appropriate measures to help them. Finland also ratified the Council of Europe Convention on the Protection of Children Against Sexual Exploitation and Sexual Abuse in 2011 (Lanzarote Convention).

CSV has been criminalized by almost every country (Convention on the Rights of the Child, n.d.). Under the Criminal Code of Finland, sexual violence against a child refers to a sexual relationship between an adult and a child under the age of 16 or an adult performing sexual acts on or touching a child in a way that harms the child’s development or causes the child to commit such acts (CCF 39/1889, n.d., 20:6). The forms of sexual violence may vary and include touching, stroking, kissing, and attempted intercourse. Sending sexually suggestive messages is also defined as sexual violence against a child (Ellonen, 2013, p. 52). The Criminal Code of Finland divides sexual violence against children into sexual violence against a child and aggravated sexual violence against a child (CCF 39/1889, n.d., 20:6, 7). The age of consent is 18 years (otherwise the age of consent is 16) in situations in which the perpetrator of sexual violence against children is, for example, the child’s teacher or other person in a position of authority (CCF 39/1889, n.d., 20:5) or in which sexual services are purchased from a young person (CCF39/1889, n.d., 20:8a). However, there is room for interpretation in defining sexual violence against a child. For example, sexual acts that take place in a consensual relationship between two minors are not defined as sexual violence in Finland.

Child welfare legislation is based on the idea that child welfare depends on all public welfare authorities and that they need to support and provide services to the families (Korpilahti et al., 2020; Pösö, 2011). In recent years, legislation has been amended to promote the investigation of sexual violence against children and to increase related penalties. The obligation to submit a child welfare notification to social services was extended to cover more professionals than previously (e.g., social, health, and educational services have had a duty to notify social services since 1984), including the Criminal Sanction Agency, fire and rescue services, Customs, the Border Guard, and the National Enforcement Authority (Section 25 of the CWA 417/2007, n.d.). For these organizations, the duty to notify social services when a child’s safety is concerned was codified in 2010 and 2014. In Finland, a child’s disclosure of CSV to a professional is always considered a formal disclosure, as all professionals working with children have had a duty to report this to social services and the police since 2011. Before that, voluntary reporting was possible. The maximum penalty for CSV was increased, and a provision for the aggravated rape of a child was added to the act in 2019 (CCF 39/1889, n.d., 20:7, 7b).

Previous studies have revealed major differences in the prevalence of CSV globally (Barth et al., 2013; Priebe & Svedin, 2008; Stoltenborgh et al., 2011). However, the findings that girls report significantly more sexual violence than boys are consistent. In surveys conducted in Europe, an average of 15.8% of girls and 3.8% of boys reported having experienced CSV (Andrews et al., 2004; Stoltenborgh et al., 2011; see also Pereda et al., 2009). According to the Finnish Child Victim Survey (FCVS), 4% of ninth graders reported experiences of CSV in 2013, 7% in 2008, and 12% in 1998. The number of both girls and boys reporting CSV has decreased during the last decades in Finland (girls—1988: 17%, 2008: 11%, 2013: 7%; boys—1988: 6%, 2008: 2%, 2013: 2%) (Fagerlund et al., 2014). Eleven percent of Finnish adult women reported having experienced sexual violence during childhood (FRA, 2014, p. 123). Differences in previous research results may stem from changes not only in the frequency of violence but also in the manifestations, identification, definition, interpretation, and reporting of violence (Mathews & Collin-Vézina, 2019).

Researchers (see, e.g., Barth et al., 2013; Edgardh & Ormstad, 2000; Fagerlund et al., 2014; Seto et al., 2015) agree that the majority of CSV victims are girls and that the majority of perpetrators are men. As a rule, in situations involving intrafamilial violence, the abusers are either fathers, stepfathers, or brothers (Seto et al., 2015). Even when the victim of CSV is a boy, the perpetrators are primarily men (Stemple et al., 2017). Therefore, CSV is a gendered phenomenon in various ways. Studies have explained the gendered nature of the phenomenon from many perspectives, linking it to broader, gendered societal and cultural, ideological, institutional, and affective practices, as well as power relations between genders and generations (Banyard et al., 2004; Cashmore & Shackel, 2014; Husso et al., 2017a; Hietamäki et al., 2021; Ward & Siegert, 2002).

## Disclosure of Sexual Violence

Studies on CSV disclosure define the term “disclosure” in different ways. For example, it is referred to as: (a) telling someone about CSV, (b) talking about it in a formal interview (e.g., making a statement to authorities, such as the police or social services), and (c) “disclosure work” occurring in therapy (Alaggia, 2004; Jones, 2000; Ullman, 2003). Furthermore, disclosure is often expressed implicitly in the literature, and there is variation depending on whether delayed disclosure and nondisclosure are differentiated (Lahtinen, 2022; McGuire & London, 2020). The definition of disclosure in the present study is described in detail in the research data and methods section.

Previous research has shown that disclosing sexual violence often takes a long time for victims (London et al., 2008; McGuire & London, 2020). The percentage of those who disclose experiences of sexual violence varies across studies. International retrospective studies report that 31% to 45% of adults have disclosed experiences of sexual violence to someone. Boys have been found to disclose it less often than girls (London et al., 2008).

According to the Well-Being of Children and Young People—School Health Promotion Study (2019) in Finland, 27% of girls and 21% of boys from a sample including eighth and ninth graders had disclosed sexual harassment or violence committed by an adult to a trusted adult. The survey did not specify the adult (Well-Being of Children and Young People—School Health Promotion Study, 2019). Similarly, Lahtinen and colleagues (2018) found that only 26% of children responding to the FCVS 2013 study had disclosed their experience to an adult (parent[s] and/or authorities), and when analyzed separately, disclosure to authorities was even rarer (12%). Disclosing experiences of CSV to a friend was the most common (48%) (see Bottoms et al., 2016). Children are notably more likely to report CSV to their peers than to adults, as reported by many studies (Bottoms et al., 2016; Helweg-Larsen & Bøving Larsen, 2006; Hietamäki et al., 2020; Lahtinen et al., 2018; Priebe & Svedin, 2008). Exposing CSV often requires that the child disclose the incident to an adult. Even if suspicion of CSV has emerged in connection with other investigations, or the authorities have been informed of it by someone else, the child may still refuse to disclose it (see, e.g., Lyon et al., 2020). The physical signs of violence alone are not enough to verify actual violence in the criminal justice process; they need to be supported by additional detailed information (e.g., disclosure of what caused the signs and symptoms) (Goodman-Brown et al., 2003).

Many victims of CSV do not disclose the incident until adulthood, which has been linked to issues such as difficulties recognizing sexual violence as something to disclose and uncertainty regarding how and to whom to disclose it (Lahtinen et al., 2018; Manay et al., 2022; McGuire & London, 2020). Studies (Husso, 2003; Laitinen, 2004; McElvaney, 2015) have shown that a lack of support, fear of negative consequences, and feelings of shame and guilt may prevent or at least delay the disclosure of CSV. Such violence occurs within and is enabled by power relationships in which an adult carries the responsibility for CSV. The exercise of power may involve persuasion, pressure, and physical violence to convince or force the child to engage in sexual interactions. These may also render the disclosure of sexual violence threatening and frightening for the child (Husso, 2003; Laitinen, 2004; McElvaney, 2015).

Other reasons victims remain silent include a lack of trust in parents (or adults in general) and a suspicion that they would not believe CSV occurred or that disclosure would result in punishment (London et al., 2008; Morrison et al., 2018; Schaeffer et al., 2011). However, the reporting of CSV by a family member can also be hindered by a sense of loyalty to the perpetrator and fear of consequences for the family (Malloy et al., 2011). Also, the closer the abuser is to the victim, the more difficult it is for the latter to disclose CSV. Experiences of violence may also remain undisclosed because no one asks about them (McElvaney, 2015). Instead, encouragement and directly asking about CSV have been found to promote disclosure in many studies (e.g., Lemaigre et al., 2017; Schaeffer et al., 2011).

Prior studies have examined several possible factors predicting disclosure, such as victim characteristics (age and gender), the offender–victim relationship, family support, and variables measuring the severity of abuse (Leclerc & Wortley, 2015). However, as earlier research (Lahtinen, 2022; Leclerc & Wortley, 2015; London et al., 2008) has pointed out, findings have been inconsistent, and more research is still needed. For example, evidence from studies with child/adolescent samples considering gender differences has been mixed, either finding boys to be more reluctant to disclose CSV (Azzopardi et al., 2019; Hershkowitz et al., 2007) or not finding a significant gender difference (Lahtinen et al., 2018; Lam, 2014; Tashjian et al., 2016). Gender differences in the disclosure patterns of CSV are rarely explored with representative adult samples (Okur et al., 2020). Okur et al. (2020), exploring both informal and formal disclosure, found that the informal disclosure rate of CSV was 2.4 times higher for female than for male victims, with the effect of gender remaining significant even after controlling for the effects of other factors; however, no significant difference between male and female participants was found in the context of formal disclosure.

The majority of previous studies on CSV disclosure have been conducted with retrospective adult samples or children interviewed in forensic or clinical contexts, often after having already disclosed CSV to someone. Population-based studies with children are still rare (Lahtinen, 2022; London et al., 2008). In this study, we combined two representative datasets to address limitations related to retrospective samples (e.g., differences between adult and child perspectives, memory limitations, and data that reflect the viewpoints of children from the past decades rather than today).

This study aimed to examine the differences between girls and boys in disclosing CSV. We examine gender differences between girls and boys in disclosing CSV and to whom they disclose it. Prior research findings have been inconsistent regarding gender differences in disclosure. Thus, no specific hypotheses were proposed. Instead, an exploratory approach was chosen. This research contributes to the previous literature on disclosing sexual violence by combining two large population-based samples of children and focusing on gender differences. Results from the FCVS have never been presented before by combining subsamples from two surveys. This allowed us to look at the gender gap, which was difficult to examine due to the extremely low frequency of CSV against boys when only one of the datasets was used (e.g., Lahtinen et al., 2022). Furthermore, combining the datasets allowed us to compare the two age groups of children in more detail.

## Research Data and Methods

The full dataset consists of data from the FCVS collected from 11 to 17 year olds in 2008 and 2013 (Hietamäki et al., 2020). The data were collected from Finnish-speaking and Swedish-speaking sixth and ninth graders from mainland Finland using an online survey (Ellonen et al., 2008b, 2013b). A stratified cluster sampling method based on county, type of municipality, and size of school was used to obtain a nationally representative sample. The participants responded to the survey at school during school hours. The research team instructed teachers on how to present the survey. Parental consent was not required. For more details on the research procedure, see Fagerlund et al. (2014). The data are a representative sample of sixth- and ninth-grade Finnish- and Swedish-speaking children in Finland and include 13,459 responses from 2008 and 11,364 responses from 2013. Half of the respondents were girls, and half were boys (2008: 50% girls, 50% boys; 2013: 51% girls, 49% boys). Eighty-eight percent of sixth graders and 64% of ninth graders participated in the study in 2008 (Ellonen et al., 2008a). In 2013, 75% of pupils responded to the survey (Fagerlund et al., 2014). According to the missing data analyses performed on the surveys, the response rate was significantly lower in the regions of Northern Finland and Lapland and Eastern Finland in 2013, which may have significantly affected the reliability of the results for CSV. Because the missing data were not completely random (Fagerlund et al., 2014, p. 29), unlike in 2008 (Ellonen et al., 2008a, p. 34), we do not make comparisons between the two datasets; however, presenting them separately is crucial to forming an overall picture and obtaining information about possible differences.

The surveys examined the children’s life situations and experiences with different forms of violence. The questions formulated by Sariola (1990) concerning CSV were repeated in later surveys with some variation (Ellonen et al., 2008a, pp. 28, 32). For the most part, data collection was carried out similarly at each measurement point. However, the survey was partly revised in 2013 (response options added in 2013 are in *italics*). In the present study, the results are presented based on the 2013 version of the survey.

As a screening question, the children were asked, “Have you any experiences of sexual advances from or sexual activity with adults or persons at least 5 years older than you?” Only those who answered “yes” saw more detailed follow-up questions related to their first experiences only. The response alternatives included in the questionnaire that were missing in the 2008 dataset are shown in italics in the following section, which examines the composite variables formed based on the variables included in the response alternatives.

Perpetrator-related questions in the survey included: “Who was that person?” The following composite variables were formed based on the responses concerning the perpetrator: (a) an unknown person (a person unknown to you), (b) a peer or sibling (your friend, boyfriend/or girlfriend, ex-boyfriend/ex-girlfriend, sister, brother, *stepsister*, *stepbrother*, or cousin), (c) a known adult (mother, stepmother, father, stepfather, grandfather, uncle or aunt, teacher, hobby instructor, *or coach*), (d) another known person (a person you knew but who was not your friend; a friend of your parents; someone else). In 2008, the respondents had the option of selecting several perpetrators, whereas in 2013, they could select only one. In the 2008 dataset, the responses containing several perpetrators were coded in such a way that “a known adult” was given priority in the coding and “another known person” was given secondary importance if the respondent had selected more than one perpetrator.

Respondents were also asked about their disclosure of sexual violence via the following item in the questionnaire: “Did you ever tell anyone about the first sexual experience you had with a person at least 5 years older than you? You can choose one or more of the following options if they apply.” The following composite variables were formed based on the alternatives: (a) no one (I did not tell anyone about it), (b) a parent (mother, father), (c) a peer or sibling (a friend, sister, or brother), and (d) an authority (the police, a school health nurse, a school social worker, a social worker). Disclosing parents, peers, or siblings refers to informal disclosing, while disclosing authorities professionals is understood here as formal disclosing. For more detailed information on the data collection, see Ellonen and Pösö (2011b), Fagerlund and colleagues (2014), and Lahtinen (2022).

The reaction to the disclosure was investigated with the question: “How did the person(s) you told react, or what did they do?” The response options were as follows (some were combined): (a) discussed with the parties involved, (b) consoled and supported me, (c) encouraged me to seek help from authorities, (d) did not support me (combined response options: did not believe me or underrated the matter, was angry with me, advised me not to talk about it to anyone), and (e) reported it to the authorities (combined response options: filed a child welfare notification, filed a criminal complaint). A further question was, “Why haven’t you told anyone?” The following were the response options (some of them were combined): (a) I didn’t think it was serious enough to tell about, (b) Did not believe that telling would help (combined response options: I didn’t dare tell, I am/was too embarrassed to tell, I don’t think anyone cares about it, I don’t think telling about it would be of any use).

The data were analyzed using IBM SPSS Statistics 25.0 software using frequency analysis, cross tabulation, and the *χ*² test. The data analysis used partial data, which included respondents under the age of 16, those who reported the perpetrator, and cases in which the perpetrator was at least 5 years older than the victim. If the respondent did not report the age of the perpetrator, the age difference was assumed to be at least 5 years (Fagerlund et al., 2014, p. 82). The aim of limiting the responses to those of children under the age of 16 was to include cases legally defined as CSV (CCF 39/1889, n.d., 20:6, 7).

Of all respondents to the 2008 and 2013 FCVS, 4.0% (*n* = 957) reported experiencing sexual advances or activity with adults or a person at least 5 years older than them. The percentage of respondents reporting experiences of CSV was higher in 2008 (4.8%) than in 2013 (3.1%). Of the respondents reporting experiences of CSV, 7.2% were girls and 2.4% were boys (*p* = .000) in 2008. In 2013, 4.4% were girls and 1.6% boys (*p* = .000).

We examined children who participated in an FCVS and experienced CSV in 2008 (*n* = 335) or 2013 (*n* = 202) (total *n* = 537). The majority of the victims were girls in 2008 (86.0%) and 2013 (84.2%). The results include cases in which the child, in response to the screening question, identified experiencing a type of sexual advance or activity.

The FCVS also contains sensitive issues related to experiences of violence. Therefore, particular attention was paid to ethics, voluntary participation, anonymity, and informing the children where to get help if they felt anxious after answering the survey. Children may be asked for the first time about their experiences of sexual violence when participating in this study, and it may be the first time for them to report their experiences (Schönbucher et al., 2012). As a result, it is also important that the survey include information about help providers (Fagerlund & Ellonen, 2016). For a more extensive reflection on the ethical issues in data collection, see Ellonen and Pösö (2011a, 2011b) and Fagerlund and Ellonen (2016).

## Results

Approximately one-third of the girls and boys who reported sexual violence responded that the perpetrator was a person unknown to them (Table 1). The second most common perpetrator type was someone the victim was familiar with, and nearly as often, it was a peer or sibling of the victim. The perpetrator was reported least frequently as being “some other known person.” The responses of girls and boys differed (*p* = .002). A larger proportion of girls than boys reported that the perpetrator was an “unknown person” or “some other known person.” Meanwhile, boys reported more often that the perpetrator was a peer or sibling or a familiar adult.

**Table 1.** Perpetrators of Sexual Violence, Girls and Boys.

Perpetrator	Year 2008						Year 2013*						Total**					
	Girl		Boy		Total		Girl		Boy		Total		Girl		Boy		Total	
	*n*	%	*n*	%	*n*	%	*n*	%	*n*	%	*n*	%	*n*	%	*n*	%	*n*	%
Unknown person	103	36	12	26	115	34	62	37	6	19	68	34	165	36	18	23	183	34*
Peer or sibling	67	23	17	36	84	25	44	26	10	31	54	27	111	24	27	34	138	26
Known adult	27	9	8	17	35	10	15	9	8	25	23	11**	42	9	16	20	58	11**
Another known person	91	32	10	21	101	30	49	29	8	25	57	28	140	31	18	23	158	29
Total	288	100	47	100	335	100	170	100	32	100	202	100	458	100	79	100	537	100

* *p* < .05; **p < .01; ***p < .001.

A quarter of the children reported not telling anyone about the CSV (Table 2). Most commonly, they disclosed it to a friend. They also disclosed it to family members, although notably less often. Among family members, CSV was disclosed to the mother slightly more often than to the father. CSV was least frequently reported to the authorities, such as social workers, the police, a school social worker, or a school health nurse.

**Table 2.** Disclosure of the Experience of Sexual Violence.

Disclosure	Year 2008						Year 2013						Total					
	Girl		Boy		Total		Girl		Boy		Total		Girl		Boy		Total	
	*n*	%	*n*	%	*n*	%	*n*	%	*n*	%	*n*	%	*n*	%	*n*	%	*n*	%
Peer or sibling	192	67	15	32	207	62***	112	70	10	36	122	65***	304	68	25	33	329	63***
Parent	56	19	10	21	66	20	47	29	6	21	53	28	103	23	16	21	119	23
Authority	23	8	3	0	26	8	21	13	6	21	27	14	44	10	9	12	53	10
No one	60	21	27	57	87	26***	36	22	7	25	43	23	96	21	34	45	130	25***
Total	288	100	47	100	335	100	161	100	28	100	189	100	449	100	75	100	524	100

* *p* < .05; **p < .01; ***p < .001.

An examination of the results on disclosure revealed that a larger share of boys than girls had not told anyone about their experience of CSV (*p* = .000). Girls reported their experiences to a peer or sibling markedly more often than boys (*p* = .000). There were no statistically significant differences between girls and boys in disclosing CSV to parents or authorities.

The perpetrator of sexual violence was reported to have been male (86%) markedly more often than female (14%). Ninety-seven percent of girls (*n* = 441) and 31% of boys (*n* = 23) (*p* = .000) reported that the perpetrator was male. Boys were more likely not to disclose a CSV experience to anyone in situations where the perpetrator was a male (56%) rather than a female (27%) (*p* = .024). Of the girls, 46% did not disclose their CSV experience to anyone when the perpetrator was a female, whereas 21% did not when the perpetrator was a male (*p* = .045).

Experiences of CSV were considerably more likely to be disclosed informally to friends, siblings, or parents than formally to authorities. CSV was most commonly disclosed informally to a peer or sibling, regardless of the perpetrator type (Table 3). The survey included options concerning both informal disclosing to peers or parents and formal reporting to the authorities. The victims were more likely to report the incident to a parent when the perpetrator was a familiar adult compared to the other perpetrator types (*p* = .000). Sexual violence was also reported formally to the authorities more often when the abuser was a known adult (*p* = .001). In situations where the child did not disclose the violence to anyone, there were no differences related to the perpetrator.

**Table 3.** The Perpetrator of Sexual Violence and Disclosure in 2008 and 2013.

Disclosed	Perpetrator									
	Unknown Person		Peer or Sibling		Known Adult		Another Known Person		Total	
	*n*	%	*n*	%	*n*	%	*n*	%	*n*	%
Peer or sibling	119	65	82	62	30	54	98	64	329	63
Parent	40	22	17	13	26	46	36	24	119	23***
Authority	15	8	9	7	14	25	15	10	53	10**
No one	46	25	39	29	12	21	33	22	130	25

* *p* < .05; **p < .01; ***p < .001.

The child’s age was associated with the disclosure of experiences of sexual violence. Forty-two percent of sixth graders and 21% of ninth graders had not told anyone about the experience (*p* = .000). In both age groups, a larger proportion of boys (63% in 6th grade and 33% in 9th grade; *p* = .006) did not disclose the CSV experience to anyone compared to girls (33% in 6th grade and 19% in 9th grade; *p* = .011). A distinctly smaller share (35%) of sixth graders than ninth graders (70%) reported the violence to a peer or sibling (*p* = .000). Sixth and ninth graders did not differ in their frequency of disclosing the incident to parents and authorities.

**Figure 1.**
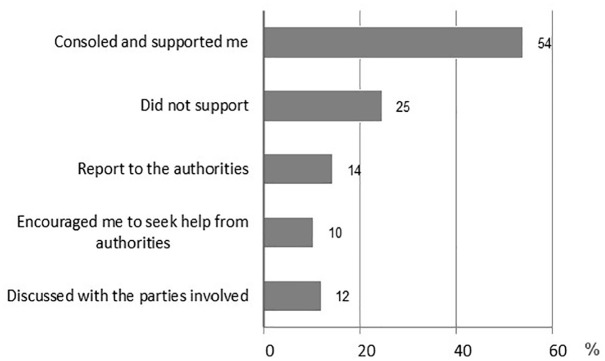
Children’s experiences of the reaction to the disclosure of sexual violence in 2013 (*n* = 126).

The reactions to the disclosure of CSV by children were diverse (girls: *n* = 109; boys: *n* = 17). More than half (54%) of the children responded that they had received comfort and support (Figure 1). A quarter of the children reported that the person they had confided in did not support them, did not believe them, got angry with them, or advised them not to talk about the matter. According to children’s responses, only in a small number of cases (14%) did the disclosure result in a formal report to authorities (police report or a child welfare notification). The children were encouraged to seek help from the authorities in one in ten cases. Based on the respondents’ reports, in a large share of the situations where the respondent had been encouraged to seek help from the authorities, the reaction had also included filing a child welfare notification and/or reporting the offense (82%).

The 2013 FCVS asked the children why they had not told anyone about the CSV incident (girls: *n* = 35; boys: *n* = 5). More than half (57.5%) who responded to the question indicated that their reason for not disclosing it was that they did not believe that doing so would be helpful. In addition, a little less than half (42.5%) of the children responded that they did not consider the matter serious enough to disclose it to anyone.

## Discussion

The results show that children are most likely to disclose CSV to peers or siblings who are considered as informal disclosure recipients. Thus, sexual violence remains largely hidden from adults—both the child’s parents and the authorities. Less than one in four children disclosed sexual violence to their own parents, and one in ten reported it to authorities. This is in line with previous studies (e.g., Lahtinen et al., 2018; London et al., 2008; Priebe & Svedin, 2008). A notably larger proportion of girls than boys disclosed CSV to a peer or sibling, but they barely differed in disclosing it to a parent or authority (see also Lahtinen et al., 2018). A quarter of children did not disclose CSV to anyone, including a considerably larger proportion of boys than girls (see also McElvaney, 2015; London et al., 2008).

Sexual violence, being subjected to sexual violence, and disclosing it as a phenomenon are variously gendered, and gender differences emerged in the data. For example, the victims of CSV were mostly girls, and the perpetrators were mainly men. Similar results have been obtained in studies conducted both in the Nordic countries and in Europe (Andrews et al., 2004; Kloppen et al., 2016). Disclosure is also partly gendered; we found that a considerably larger proportion of boys than girls had not disclosed CSV to anyone. Other studies have also found that girls report sexual violence more often and sooner after it has occurred than boys (e.g., Goodman-Brown et al., 2003; Schaeffer et al., 2011; Priebe & Svedin, 2008). The results of this study indicate that the gender of the perpetrator was also associated with the disclosure of CSV in both girls and boys. Both were less likely to report it when the perpetrator was of the same gender as the victim. Previous studies have found that in the case of boys who have been subjected to CSV, a fear of being labeled homosexual may prevent or delay disclosure (Lemaigre et al., 2017; McElvaney, 2015).

In the present study, the victim–perpetrator relationship was also related to the person to whom the child disclosed CSV. The perpetrator being a known adult increased the likelihood of disclosure to a parent or an authority. However, they disclosed the violence to a friend equally often, regardless of the perpetrator. Similarly, regardless of how familiar the perpetrator was, approximately as large a proportion of the children did not disclose their experience to anyone. This indicates how important it is to accurately define what is meant by “disclosure,” and whether it means informal or formal disclosing. Variables predicting disclosure may vary depending on the context of the disclosure. This may also explain the mixed findings for the effect of the offender–victim relationship on disclosure in previous studies (for a review, see London et al., 2008).

The child’s age was also linked to the disclosure of a CSV experience. In line with earlier studies (Alaggia et al., 2019; McElvaney, 2015), a larger share of the sixth-grade than ninth-grade children replied that they had not told anyone about a CSV incident. An age difference was also found in disclosure to peers or siblings; notably, more ninth graders than sixth graders reported disclosing a CSV incident to peers or siblings. However, no difference between sixth and ninth graders was found in disclosure to parents or authorities. These findings again show that the effect of the age variable may be dependent on the context of disclosure.

Children’s self-reported reasons for nondisclosure in this study, which combined data from two FCVS surveys, were in line with Lahtinen et al. (2018), who analyzed 2013 FCVS data, finding that more than half of the children responded that they had been too afraid or ashamed to disclose their CSV experiences, that they believed no one would be interested, or that disclosure would not be helpful. Slightly fewer than half of the children reported that they had not considered the incident serious enough to disclose it. More than half of the children who reported their experience and its consequences felt comforted and supported. However, one in four replied that the reporting had resulted in negative reactions. They felt that they had not been believed, their experiences were downplayed, or the person in whom they confided became angry with them or told them not to speak about their experiences again. Only one in ten had been encouraged to seek help, and a police report or child welfare notification had been filed for just over one in ten cases. A low level of support, fear of negative consequences, and feelings of shame and guilt have been shown to prevent the reporting of violence (Morrison et al., 2018; Schaeffer et al., 2011). Experiences of CSV may also remain undisclosed because no one asks about them (McElvaney, 2015; Morrison et al., 2018). Accordingly, factors promoting disclosure include directly asking the child about violence, raising awareness of sexuality and sexual violence, and providing children with a safe atmosphere and an opportunity for confidential discussions (Alaggia et al., 2019).

Remaining silent about sexual violence continues to be common in our culture. The disclosure of sexual violence is also prevented by feelings of shame that silence victims of violence, as well as by a tradition of blaming the victims of gender-based and sexual violence (Husso et al., 2017b, 2021). Gendered assumptions and expectations concerning girls and boys also maintain and shape the disclosure of violence or remaining silent, leading to gendered customs and practices (Alaggia et al., 2019; Husso, 2016). Encountering, identifying, naming, and verbalizing experiences is often challenging for adults, which, in turn, affects children’s willingness and opportunities to disclose violence (Husso et al., 2017b; Laitinen, 2004). Whether people report experiences of violence depends essentially on their reasons for remaining silent and the type of threat that revealing the matter poses to victims and their loved ones. The attitudes and reactions of those in whom the child confides, such as parents, authorities, and professionals, also significantly affect the child’s recollection and disclosure of experiences of violence (Gemara et al., 2023).

The failure to disclose and report sexual violence has far-reaching consequences in today’s society, where symbolic and written documents constitute our conceptions of the reality (Ferraris, 2012; Husso et al., 2014, 2021). When CSV is not disclosed, reported, or considered, we are unable to understand how frequent violence is and the extent of the damage it causes. This highlights the importance of addressing violence against children, CSV, or related suspicions, and for professionals not to hesitate to ask directly about them.

### Strengths and Limitations

The data consist of a representative sample of children in Finland (*N* = 13,459 for 2008; *N* = 11,364 for 2013). The combined dataset made it possible to examine the victims and perpetrators of CSV and its reporting by gender in Finnish data. However, there was a minor change in screening questions related to sexual advances in the different data collections in Finnish but not in English. Thus, the respondents may have understood and interpreted the questions in various ways. Not all the children might have noticed, identified, or understood the screening question and might, therefore, not have responded to the follow-up questions. Another limitation is that a peer or sibling as perpetrator of CSV included several options, which were your friend, boyfriend/girlfriend, ex-boyfriend/ex-girlfriend, sister, brother, stepsister, stepbrother, or cousin. These options are highly variable and are not necessarily peers or siblings for the victim. Moreover, the examined data do not include information about CSV that occurs via digital media. In the 2013 dataset, the children were asked about meeting someone via contact initiated online and subsequent CSV, but the form did not contain a question about the disclosure of experiences of violence. Due to the lack of examination of the forms of digital violence, it is likely that the results do not provide comprehensive information about CSV and its disclosure. In addition, we were unable to consider diverse groups, gender minorities, and other minorities, in this study, no response options were provided for gender other than “girl” or “boy” in the 2008 of 2013 FCVS, and frequencies of people with foreign backgrounds were low. Finally, as we did not conduct multivariate analyses, the effects of other variables on disclosure could not be considered. Therefore, more research is needed on the independent effects of gender and age when the effects of other variables are excluded.

## Conclusions

From the perspective of enabling societal change, special attention should be paid to gender-sensitive activities that promote disclosure. When developing measures that ease preventive work and disclosure, it is crucial to focus on issues such as the gendered nature of sexual violence, disclosure of experiences of violence, younger children’s tendency to remain silent about such experiences, the tendency to stay silent about violence by a perpetrator of the same gender, and the role of children’s peers or siblings as supportive recipients of disclosure who can also offer support in disclosure to adults.

Disclosure rates of CSV and other forms of violence, as well as listening to the victims, have not only individual but also legal and political impacts. Such disclosure is essential to both recovery and preventing further incidents (Lemaigre et al., 2017; McElvaney et al., 2012; Schaeffer et al., 2011). Identifying the experiences of abused children, taking them seriously, and interventions against CSV are prerequisites for the broader development of the practices and operating culture of child and family work, along with the changes in attitudes and structures they require. This would save human suffering, the need for services, and social costs in the long term, extending into adulthood (e.g., Siltala et al., 2022).
